# Asymptomatic bacteriuria, antibiotic use, and suspected urinary tract infections in four nursing homes

**DOI:** 10.1186/1471-2318-12-73

**Published:** 2012-11-23

**Authors:** Charles D Phillips, Omolola Adepoju, Nimalie Stone, Darcy K McMaughan Moudouni, Obioma Nwaiwu, Hongwei Zhao, Elizabeth Frentzel, David Mehr, Steven Garfinkel

**Affiliations:** 1 Texas A&M Health Science Center, 1266 TAMU, College Station, TX, 77843, USA; 2 Centers for Disease Control and Prevention, 1600 Clif2009ton Road, Atlanta, GA, 30333, USA; 3 American Institutes for Research, 100 Europa Drive, Suite 315, Chapel Hill, NC, 27517, USA; 4 University of Missouri-Columbia School of Medicine Curtis W. and Ann H. Long Dept. of Family and Community Medicine, M226 Medical Sciences, Columbia, MO, 65212, USA

**Keywords:** Nursing home, Antibiotic stewardship, Urinary tract infection, Asymptomatic bacteriuria, Antibiotics

## Abstract

**Background:**

Urinary tract infections (UTIs) are the most commonly treated infection among nursing home residents. Even in the absence of specific (e.g., dysuria) or non-specific (e.g., fever) signs or symptoms, residents frequently receive an antibiotic for a suspected infection. This research investigates factors associated with the use of antibiotics to treat asymptomatic bacteriuria (ASB) among nursing home residents.

**Methods:**

This was a cross-sectional study involving multi-level multivariate analyses of antibiotic prescription data for residents in four nursing homes in central Texas. Participants included all nursing home residents in these homes who, over a six-month period, received an antibiotic for a suspected UTI. We investigated what factors affected the likelihood that a resident receiving an antibiotic for a suspected UTI was asymptomatic.

**Results:**

The most powerful predictor of antibiotic treatment for ASB was the presence of an indwelling urinary catheter. Over 80 percent of antibiotic prescriptions written for catheterized individuals were written for individuals with ASB. For those without a catheter, record reviews identified 204 antibiotic prescriptions among 151 residents treated for a suspected UTI. Almost 50% of these prescriptions were for residents with no documented UTI symptoms. Almost three-quarters of these antibiotics were ordered after laboratory results were available to clinicians. Multivariate analyses indicated that resident characteristics did not affect the likelihood that an antibiotic was prescribed for ASB. The only statistically significant factor was the identity of the nursing home in which a resident resided.

**Conclusions:**

We confirm the findings of earlier research indicating frequent use of antibiotics for ASB in nursing homes, especially for residents with urinary catheters. In this sample of nursing home residents, half of the antibiotic prescriptions for a suspected UTI in residents without catheters occurred with no documented signs or symptoms of a UTI. Urine studies were performed in almost all suspected UTI cases in which an antibiotic was prescribed. Efforts to improve antibiotic stewardship in nursing homes must address clinical decision-making solely on the basis of diagnostic testing in the absence of signs or symptoms of a UTI.

## Background

Urinary tract infections (UTIs) are the most commonly treated infection among nursing home residents [1-5]. In a recent prevalence survey of infections in U.S. Department of Veterans Affairs long-term care facilities, symptomatic UTIs accounted for 29% of all infections among nursing home residents [6]. However, accurate diagnosis of a UTI poses significant and distinctive challenges in the nursing home setting. Much of this difficulty arises due to the prevalence of asymptomatic bacteriuria (ASB) among nursing home residents. ASB is common in the resident population, with prevalence sometimes as high as 50% [7,8]. Residents with ASB have no specific (e.g., dysuria) or non-specific (e.g., fever) signs or symptoms of a UTI. However, urinalyses performed on these residents will exhibit abnormalities due to the presence of bacterial in the urine [9]. Therefore, residents without physical signs or symptoms of a UTI but with abnormal urine study results may receive a course of antibiotics due to ASB.

Antibiotic overuse for ASB occurs even though no evidence indicates that treating ASB in older adults is of benefit. Antimicrobial treatments do not affect the prevalence of bacteriuria, the frequency of symptomatic urinary tract infections, morbidity, or mortality [10-14]. Moreover, such treatments are potentially harmful. Antibiotic use in nursing homes is a strong driver for the emergence of multi-drug resistant organisms (MDROs) such as methicillin-resistant *Staphylococcus aureus* (MRSA), vancomycin-resistant *Enterococci* (VRE), and fluoroquinolone-resistant gram negative bacilli [15-21]. In addition, in an investigation in two Rhode Island nursing homes, residents with ASB who were treated with an antibiotic were 8.5 times more likely to develop *Clostridium difficile* within the three months following their course of antibiotics [22].

To assist clinicians in differentiating symptomatic UTIs from ASB, several consensus guidelines have been developed that provide criteria for diagnosis and management of a suspected UTI in a nursing home resident [23,24]. In these guidelines, the presence of signs and symptoms localizing to the urinary tract, such as dysuria and suprapubic discomfort, are important criteria for diagnostic evaluation and the decision to implement antibiotic treatment for a suspected UTI.

As part of a larger study of antibiotic use in nursing homes, we investigated antibiotic use among residents in four nursing homes who were treated with an antibiotic for a suspected UTI. In this cohort of nursing home residents receiving antibiotics for a suspected UTI, our study investigated differences between residents treated with an antibiotic who presented with documented signs or symptoms of a UTI and those who received an antibiotic but were asymptomatic.

## Methods

### Data

A retrospective chart review was performed to obtain data from the four study nursing homes about nursing home residents receiving antibiotics for a suspected UTI. Data collection was part of a larger study on antibiotic stewardship in nursing homes. The four study facilities, all located in central Texas, ranged in size from 48 to 222 beds and included for-profit homes, not-for-profit homes, and homes that are part of multi-facility systems.

From April 2010 through September 2010, residents receiving an antibiotic for a suspected UTI were identified from the four study homes’ infection logs, which list all infections in the home and which federal regulations require homes to maintain [56 FR 48876, Sept. 26, 1991, as amended at 57 FR 43925, Sept. 23, 1992]. All but a scant few of the listed infections were associated with an antimicrobial prescription, so that the infection logs essentially represent antibiotic treatment logs. For each identified episode of antibiotic treatment for a suspected UTI, the research team collected resident-level data, including resident characteristics at admission and information from the Minimum Data Set (MDS) assessment completed prior to the date of the prescription that brought them into our sample [25].

Additional information was gathered from the review of residents’ medical records, including nursing notes and clinician’s orders. The information in these sources included the symptoms noted at the time of the suspected infection and the prescribed treatments. Data were collected by research consultants with clinical experience in nursing homes, who were employed by the TMF Healthcare Quality Institute, the Medicare Quality Improvement Organization (QIO) for Texas. The Institutional Review Boards at Texas A&M University and the American Institutes for Research approved the on-site data collection procedures for this study. Both boards gave the research team a waiver of written consent and a waiver of consent prior to accessing Private Health Information because no personal identifiers were included in the database.

### Measurement

#### Dependent variable

The dependent variable in our analyses was a binary variable reflecting the presence or absence of any signs or symptoms associated with a symptomatic UTI, based on consensus criteria previously published by Loeb et al. (Loeb criteria) [24]. The review of medical records focused heavily on looking for documentation of the following signs and symptoms noted by the consensus panel:

•acute dysuria,

•fever (>37.9°C [100°F]) or 1.5°C [2.4°F] increase above baseline temperature),

•new or worsening urgency, frequency, or incontinence,

•suprapubic pain,

•gross hematuria,

•costovertebral angle (flank) tenderness,

•rigors, or

•delirium (recent and abrupt change in mental status).

The dependent variable equaled zero when any of the signs or symptoms above were noted in the medical record (i.e., symptomatic UTI), and it equaled one when none of these symptoms were present (i.e., ASB).

#### Independent variables

Resident characteristics were obtained using a resident’s admission MDS (version 2.0) and the most recent MDS completed prior to the date of the first suspected UTI. Information on physical functioning and cognitive patterns were used to construct established scales for activities of daily living and cognitive performance. Our model also included an indicator for potential depression, and an indicator of the presence of a problem or condition that might result relatively soon in a health decline or in death.

We investigated the effects of individual health status measures on the likelihood a prescription would be written for ASB. Although the results of previous research on the impact of functional status or case-mix on certain aspects of antibiotic use is somewhat mixed [26,27], we included a measure of functional status in our analyses. The ADL Hierarchy Scale uses residents’ performance in activities of daily living to group them according to stages in the disablement process (range from 0 to 6) [28]. Higher scores indicate greater needs for assistance to overcome activity limitations. The Cognitive Performance Scale (CPS) combines information on memory impairment, communication problems, executive function, and independence in daily decision making. The scores range from 0 (intact) to 6 (very severe impairment). Previous research has shown that the CPS is highly correlated with the Mini-Mental Status Examination [29-33] and has been used extensively for over a decade of research in nursing homes [34]. Behavioral and cognitive symptoms of depression may overlap with those of delirium in the elderly, increasing the challenge of diagnosing symptomatic UTIs. As an indicator of potential depression symptoms, we used items from the MDS Depression Rating Scale (DRS) [35]. The original DRS consists of seven items generating a score ranging from 0 (depression not exhibited) to 14 (depression exhibited daily). In our sample, the prevalence of the symptoms included in the DRS was relatively low. This made it impossible to create a full DRS with a reasonable distribution. Instead we used the DRS variables to create a dichotomous variable – the depression item - representing the presence or absence of any symptoms of depression.

Resident demographic characteristics were included in the analysis to explore any potential differences in the presence or absence of signs and symptoms for UTIs associated with age, gender and race/ethnicity. Age was categorized into 3 groups (under 85, 85+, and missing). Given the preponderance of non-Hispanic White residents and missing observations, race/ethnicity was categorized as three variables representing non-Hispanic White, all other races/ethnicities, and missing observations. A binary variable identifying any resident with multiple courses of antibiotics for a suspected UTI was also developed. Similarly, an indicator representing the nursing home within which the suspected infection took place was included in our analyses. General data on the four nursing homes were also collected by the research consultants.

### Statistical analyses

Data were analyzed using SAS 9.2. We present descriptive data for the four homes and compare baseline characteristics of all non-catheterized residents who received antibiotics for a suspected UTI, categorizing those residents by whether or not they presented with signs or symptoms thought to warrant antibiotic use.

One of the major predictors of the use of an antibiotic for a suspected UTI was the presence of an indwelling urinary catheter [24]. Data for those sixteen individuals with an indwelling catheter, who had 23 prescriptions for a suspected UTI, were analyzed separately from the data for those with no indwelling urinary catheter.

While the descriptive analysis focused on 151 individual residents without an indwelling catheter, the multivariate analysis was performed on 204 prescriptions for a suspected UTI for these 151 individuals. The unit of analysis for our multivariate modeling was the prescription. As our results indicate, a reasonable proportion of residents received more than one antibiotic for a suspected UTI during the study period. Very few of these were prescriptions were for a persistent UTI. We identified all prescriptions written for the same person over a period of one month. We then removed these individuals from our analyses. The results did not change, so these cases remain in the data used in our analyses.

Due to the correlated nature of the prescription data, in which a resident may have had more than one episode of a suspected UTI during the study period, a generalized linear mixed effect model was used to estimate the likelihood of a resident having none of the signs or symptoms identified in the Loeb criteria among those who received an antibiotic prescription for a UTI [24]. Residents’ project - assigned identification numbers were included in the model as a random effect. The fixed effects included all the independent variables mentioned earlier. The quadrature method from the GLIMMIX procedure in SAS was used for fitting the regression model. The association between each independent variable and lack of the symptoms of a UTI was expressed as odds ratio estimates, and the 95% confidence intervals for odds ratios were also obtained.

## Results

Twenty-three prescriptions in the study period were written for individuals with an indwelling catheter. Of these, 82.6 percent (19) were written when the resident displayed no localized or proximal symptoms of a UTI. Since the relative number of prescriptions was low, but the percent of prescription written for residents who were asymptomatic was so different from that for other residents, we did not use these cases in our further analyses. While recognizing the overwhelming role of an indwelling urinary catheter in antibiotic prescription for a suspected UTI, our analyses focus on those factors that result in treatment for ASB among residents who are not catheterized; the results discussed below involve only the prescriptions written for those residents without an indwelling catheter.

Table 1 reveals that facility characteristics differed among the four nursing homes involved in the study. The study included one smaller home, a much larger home, and two homes of roughly average size. Two to six prescribing clinicians served the residents in each of these homes. Clinicians ordered urine studies on most cases treated for a suspected UTI (71% to 97%). In a majority of instances, these results were available to the clinician prior to the order for a course of antibiotics (64% to 85%). Clinicians in our four study homes prescribed antibiotics for a suspected UTI at rates ranging from 2.05 to 3.32 prescriptions per 1000 resident days. The homes varied considerably in the proportion of residents for whom the medical director was the attending physician (36% to 72%) and in the percent of antibiotics (25% to 58%) prescribed for a suspected UTI when the resident presented with no documented signs or symptoms of a UTI (e.g., pain, fever).

**Table 1:** Antibiotic use for suspected UTI in four homes and home characteristics (n=204 prescriptions)

**Characteristics**	**Home A**	**Home B**	**Home C**	**Home D**
	**(N=19)**	**(N= 63)**	**(N=41)**	**(N=104)**
Antibiotic used for ASB	32%	45%	25%	58%
Urine studies performed	95%	71%	94%	97%
Lab results received prior to prescription (Rx)	84%	59%	64%	85%
Antibiotic Rx rate for Suspected UTI per 1000 resident days	2.79	3.32	2.05	2.77
No. of Beds	48	125	120	222
No. of Attending MDs	6	5	2	3
Residents Medical Director Attending	72%	36%	71%	52%
Ownership	For profit - Partnership	For profit - Individual	For profit - Corporation	Non profit—Church related

As the results in Table 2 indicate, residents in the sample displayed characteristics similar to national data from the National Nursing Home Survey [36]. The majority of residents in this study were over 85 years of age, female, and White. The average score on the ADL Hierarchy indicates these residents needed extensive hands-on assistance with ADLs such as personal hygiene and dressing and limited hands-on assistance with ADLs such as transfer and locomotion. The average CPS score was 2.0, indicating that the average resident was moderately cognitively impaired. Almost forty percent of residents exhibited some symptom of depression. Twenty six percent of residents received multiple courses of antibiotics.

**Table 2:** Baseline characteristics of residents receiving at least one course of antibiotics for a suspected UTI

**Resident characteristics, percent or mean (S.D.)**	All residents with suspected UTI (N=151)**	Asymptomatic (N=71)**	One or more symptoms of UTI (N=80)**
Age			
Under 85	35%	37%	34%
85+	56%	55%	56%
Missing	9%	8%	10%
Female	82%	85%	80%
Race			
White	72%	70%	74%
Others	7%	8%	5%
Missing	21%	21%	21%
Activities of Daily Living Hierarchy (0–6)	2.0(1.2)	2.1(1.2)	1.9(1.2)
Cognitive Performance Scale (0–6)	2.0(0.6)	2.0(0.6)	2.0(0.6)
Any symptoms of depression	39%	48%	31%
Multiple courses of antibiotic for UTIs	26%	27%	25%

*None of the differences between these two groups were statistically significant.

**There were some differential in missing observations among the variables

(April-Sept 2010)*.

Table 3 indicates that among the 204 antibiotic prescriptions written for the 151 uncatheterized residents treated for a suspected UTI, no significant differences were observed in antibiotic selection or duration of therapy between the residents with or without signs or symptoms of a UTI. The medications most often prescribed were fluoroquinolones (28% of all prescriptions) or nitrofurantoin (25% of all prescriptions). Sulfonamides, penicillins, and cephalosporins each accounted for roughly 8 to 14 percent of the medications prescribed. The average course of antibiotics lasted 7.6 days for asymptomatic cases. Urine studies were ordered in most cases where a prescription was written (89%). The most common order was for both a urinalysis and a culture with sensitivity, although a small number of orders asked for only one of these studies. In addition, urinalysis results were usually available to the prescribing clinician prior to ordering the antibiotic.

**Table 3:** Antibiotic and laboratory use when antibiotics were prescribed for a suspected urinary tract infection (n= 204)*

**Characteristics**	**Asymptomatic prescription for UTI (N=95)**	**One or more symptoms of UTI (N=109)**
Urine studies performed	89%**	89%
Lab results received prior to antibiotic prescription (Rx)	80%	69%
Antibiotics Used		
Fluoroquinolones	28%	36%
Nitrofurantoin	25%	19%
Sulphonamides	13%	10%
Penicillins	14%	10%
Cephalosporins	8%	10%
Others	12%	15%
Average days of antibiotic prescription (standard deviation)	7.6(2.20)	8.1(2.92)

*Since this analysis is based on prescriptions, some residents appear in both categories. Our analyses of differences took this into account. None of the differences were statistically significant.

**Facility records identified all prescriptions as given for UTI treatment but chart review did not find documentation of laboratory results.

In a multivariate analysis (Table 4) of the likelihood that a prescription for an antibiotic to treat a suspected UTI was written for a resident with ASB, only the site of care (nursing home) had a statistically significant effect, after adjusting for the effects of resident characteristics. Of those for whom a course of antibiotics was prescribed for a suspected UTI, residents in Home C (p=.01) were less likely to be asymptomatic compared with residents in Home D. The odds-ratio for Home A bordered on statistical significance (p=.07). Given the small sample size, we consider this difference meaningful. The proportion of antibiotics prescribed for asymptomatic residents was similar for Home B and Home D. In addition, the random effect for individuals was not significant.

**Table 4:** Estimates of covariate effects on the likelihood that an antibiotic prescription was for asymptomatic resident (n = 204)

**Independent variables**	**Odds ratios**	**95% confidence intervals**
Activities of Daily Living Hierarchy	1.02	(0.76,1.37)
Cognitive Performance Scale	0.80	(0.43, 1.5)
Any depressive symptoms	1.36	(0.92, 2.01)
Female	1.56	(0.63, 3.87)
Lab results received prior to prescription (Rx)	1.67	(0.71, 3.94)
Age		
Under 85	1.15	(0.52, 2.58)
Missing	0.39	(0.09,1.64)
Race		
White (reference)	-	-
Others	1.32	(0.4,4.29)
Missing	1.04	(0.34,3.18)
Homes		
Home A	0.27	(0.07,1.07)
Home B	0.76	(0.32,1.8)
Home C	0.20	(0.06,0.67)
Home D (reference)	----	----
Person (random effect)	0.3495 (Variance Estimate)	0.8242 (Std. Error)

## Discussion

One of the major issues in antibiotic stewardship in nursing homes is the use of antibiotics to treat suspected UTIs [37-40]. Despite extensive research demonstrating a lack of benefit and a potential for harm for antibiotic use for ASB [9,18,41], this practice continues to be prevalent among clinicians serving nursing home residents [2,42]. This study found a substantial proportion (50%) of antibiotics prescribed for a suspected UTI was given to asymptomatic residents. Our results were also consistent with previous research that identified fluoroquinolones as the most common type of antibiotic used to treat UTIs [37,43].

Somewhat surprisingly, our results indicated that resident demographics or indicators of health and functional status did not affect the likelihood of receipt of an antibiotic despite the absence of physical symptoms for UTI. Several resident conditions such as diabetes, stroke and causes of urinary tract dysfunction were not included in these data. Nonetheless, these results imply that differences in health and functional status among residents are not a driving force behind prescribing antibiotic treatment for ASB.

Our results provide evidence of the overwhelming importance of the presence or absence of an indwelling urinary catheter in the use of antibiotics for ASB. Our results from these four homes indicate that over 80 percent of the antibiotics prescribed for individuals with a catheter were written in the absence of signs or symptoms of a UTI. However, they were usually written in the presence of urinalysis results.

Previously published infection surveillance criteria for UTIs in long term care facilities have given too little attention to laboratory data as part of the diagnostic paradigm [44]. However, in these data, we found widespread use of urine testing in the absence of signs and symptoms recognized in clinical guidelines as indicating the presence of a UTI. These results suggest that diagnostic testing may play a major role in the use of antibiotics for ASB.

Our data highlight the need for further assessment of how the interpretation of these tests might drive prescribing decisions in this setting. The medical literature emphasizes that older persons with a UTI may present differently than younger persons [45-47]. Thus, a wide range of events may prompt the ordering of urinalyses - any change in cognitive status, change in behaviors, changes in the color or smell of urine, or even a fall [48]. For cognitively impaired residents with limited communication abilities, any signs of discomfort or functional change may result in a urinalysis [49]. However, given the prevalence of ASB, there is a high likelihood that any urinalysis will be abnormal and subsequent culture positive.

Given the receipt of a positive urinalysis, the attending clinician is faced with what can be viewed as a short and long-term risk assessment decision. For the clinician the calculation of risk can be almost entirely short-term. In terms of short-term risks, by not prescribing an antibiotic, the clinician may fail to treat a blossoming infection; the resident’s condition may worsen, possibly dramatically. The benefits of not prescribing an antibiotic, such as reduction in emergence of antibiotic-resistant pathogens or avoidance of adverse drug complications’ will usually be seen in rates for the entire population in the home (e.g. decreases in *C. difficile* rates in a facility over time). Thus, the value of good antibiotic stewardship may go unrecognized by the clinician who focuses on residents sequentially, rather than as a part of a population.

Further studies should explore how the level of engagement and types of authority wielded by nursing home clinicians in homes of different sizes may influence antibiotic prescribing practices. The longer term benefits of antibiotic stewardship may be more evident to nursing home clinicians with greater responsibility in the home. If that is the case, it may provide guidance to those seeking avenues to address effectively the overuse of antibiotics and the lack of good antibiotic stewardship in long-term care.

Our study has several limitations. Our sample was based on a log of antibiotic prescriptions driven by clinical suspicion of a UTI. We did not have a comparison of this cohort to residents in the facility who did not receive antibiotics, nor could we identify individuals who had ASB but were not suspected of having a UTI. Therefore, we could not provide a comparison between individuals treated with an antibiotic for ASB and individuals with ASB not treated with antibiotics, to explore risk factors related to receiving an antibiotic. Our definition of symptomatic UTI was based on criteria developed by a consensus panel of experts, with limited validation, so some may disagree with the criteria applied in this study. Our data came from homes that varied on a number of dimensions, but all were located in central Texas, potentially affecting whether these findings are more broadly representative of the nursing home setting. Finally, data collection was retrospective and dependent on the quality of residents’ medical records in nursing homes.

## Conclusions

Despite its limitations, this study offers some interesting insights into antibiotic use in nursing homes in the management of a suspected UTI in the presence or absence of signs and symptoms. Our results indicate that further study might do well to focus on the use and interpretation of laboratory testing in clinicians’ decisions to prescribe an antibiotic for a suspected UTI. Given our findings related to the importance of the site of care, additional efforts might focus on improving knowledge of the risks and benefits of antibiotic use for ASB among nursing home staff and administration.

## Abbreviations

UTI: Urinary tract infections; ASB: Asymptomatic bacteriuria; MDROs: Multi-drug resistant organisms; MRSA: Methicillin-resistant *Staphylococcus aureus*; VRE: Vancomycin-resistant *Enterococci*; MDS: Minimum data set; QIO: Quality improvement organization; ADL: Activities of daily living; CPS: Cognitive performance scale; DRS: Depression rating scale.

## Competing interests

The authors indicate that they have no competing interests.

## Authors’ contributions

CP, EF, NS and SG planned the project. DKM, HZ, OA, ON, and were responsible for data management and data analyses. All authors were involved in reviewing and interpreting the results of the analyses. CP wrote the first draft of the article. All authors reviewed and commented on the draft. NS had lead responsibility for later drafts. All authors have read and approved the final manuscript.

## Authors’ information

CP is a Regents Professor at the Texas A&M Health Science Center (HSC) in College Station, TX. DKM is an assistant professor in Health Policy and Management at the HSC. OA and ON are doctoral students at the HSC. HZ is an associate professor of biostatistics at the HSC. DM is William C. Allen Professor in Family and Community Medicine at the University Of Missouri School Of Medicine in Columbia, MO. NS is the medical epidemiologist for long-term care in the Division of Healthcare Quality Promotion at the Centers for Disease Control and Prevention in Atlanta, GA.

## Pre-publication history

The pre-publication history for this paper can be accessed here:

http://www.biomedcentral.com/1471-2318/12/73/prepub
